# Performance of Surgical Risk Scores to Predict Mortality after Transcatheter Aortic Valve Implantation

**DOI:** 10.5935/abc.20150084

**Published:** 2015-09

**Authors:** Leonardo Sinnott Silva, Paulo Ricardo Avancini Caramori, Antonio Carlos Bacelar Nunes Filho, Marcelo Katz, João Carlos Vieira da Costa Guaragna, Pedro Lemos, Valter Lima, Alexandre Abizaid, Flavio Tarasoutchi, Fabio S. de Brito Jr

**Affiliations:** 1 Pontifícia Universidade Católica, Porto Alegre - Brazil; 2 Hospital Israelita Albert Einstein, São Paulo - Brazil; 3 Instituto do Coração, São Paulo - Brazil; 4 Hospital Santa Casa de Misericórdia, Porto Alegre - Brazil; 5 Instituto Dante Pazzanese de Cardiologia, São Paulo - Brazil

**Keywords:** Risk Factors, Probability, Aortic Valve Stenosis / surgery, Transcatheter Aortic Valve Replacement

## Abstract

**Background:**

Predicting mortality in patients undergoing transcatheter aortic valve implantation (TAVI) remains a challenge.

**Objectives:**

To evaluate the performance of 5 risk scores for cardiac surgery in predicting the 30-day mortality among patients of the Brazilian Registry of TAVI.

**Methods:**

The Brazilian Multicenter Registry prospectively enrolled 418 patients undergoing TAVI in 18 centers between 2008 and 2013. The 30-day mortality risk was calculated using the following surgical scores: the logistic EuroSCORE I (ESI), EuroSCORE II (ESII), Society of Thoracic Surgeons (STS) score, Ambler score (AS) and Guaragna score (GS). The performance of the risk scores was evaluated in terms of their calibration (Hosmer–Lemeshow test) and discrimination [area under the receiver–operating characteristic curve (AUC)].

**Results:**

The mean age was 81.5 ± 7.7 years. The CoreValve (Medtronic) was used in 86.1% of the cohort, and the transfemoral approach was used in 96.2%. The observed 30-day mortality was 9.1%. The 30-day mortality predicted by the scores was as follows: ESI, 20.2 ± 13.8%; ESII, 6.5 ± 13.8%; STS score, 14.7 ± 4.4%; AS, 7.0 ± 3.8%; GS, 17.3 ± 10.8%. Using AUC, none of the tested scores could accurately predict the 30-day mortality. AUC for the scores was as follows: 0.58 [95% confidence interval (CI): 0.49 to 0.68, p = 0.09] for ESI; 0.54 (95% CI: 0.44 to 0.64, p = 0.42) for ESII; 0.57 (95% CI: 0.47 to 0.67, p = 0.16) for AS; 0.48 (95% IC: 0.38 to 0.57, p = 0.68) for STS score; and 0.52 (95% CI: 0.42 to 0.62, p = 0.64) for GS. The Hosmer–Lemeshow test indicated acceptable calibration for all scores (p > 0.05).

**Conclusions:**

In this real world Brazilian registry, the surgical risk scores were inaccurate in predicting mortality after TAVI. Risk models specifically developed for TAVI are required.

## Introduction

Aortic stenosis, the most common acquired valvular disease, is present in 4.5% of the population aged > 75 years^1^. For patients with severe symptomatic aortic stenosis, surgical aortic valve replacement (SAVR) is considered the therapy of choice^2^. Transcatheter aortic valve implantation (TAVI) has been consolidated in recent years^3^. Initially introduced for patients deemed inoperable^4,5^, TAVI has been widely used as an alternative to surgical treatment for patients considered at a high surgical risk^6^. Surgical risk assessment in patients with severe aortic stenosis plays an important role in the selection of the best therapeutic strategy.

The mortality rates associated with SAVR can be predicted by scores that consider the preoperative characteristics of the patients. EuroSCORE^7,8^ and the Society of Thoracic Surgeons (STS) score^9^ are the most often used scores for this purpose because they have been extensively validated. Other scores such as the Ambler score^10^ and the Guaragna score^11^ have also been used, particularly for predicting the mortality of valvular heart surgery.

The currently available risk scores were designed and validated in populations undergoing coronary artery bypass graft surgery (CABG), surgical valve replacement, or combined surgery. Little is known about the usefulness of these scores to predict the mortality in patients undergoing TAVI. To date, there is no specific well-established risk score for predicting mortality in patients undergoing TAVI.

Therefore, the objective of the present study was to evaluate the performance of the established surgical risk scores to predict mortality in patients participating in a TAVI real-world registry^12^.

## Methods

In a nationwide registry conducted by the Brazilian Society of Interventional Cardiology, centers with ≥ 3 valve implantations (18 centers) were invited to participate. From January 2008 to January 2013, 418 consecutive patients undergoing TAVI were included.

The logistic EuroSCORE I^7^ (http://www.euroscore.org/calcold.html) and STS score^11^ (http://riskcalc.sts.org/de.aspx) were prospectively calculated at the time of patient inclusion, while the EuroSCORE II^8^ (http://www.euroscore.org/calc.html), Ambler score^10^ (http://www.ucl.ac.uk/statistics/research/riskmodel/index.html), and Guaragna score^11^ were calculated on the basis of the data collected during the study. All scores were developed as the predictors of in-hospital mortality.

The clinical outcomes in the study were defined by the Valve Academic Research Consortium-II (VARC-II) criteria^13^. In this analysis, the following outcomes were assessed: 30-day mortality, immediate procedural mortality (resulting from periprocedural events leading to death within 72 h after the procedure), and procedural mortality (all-cause mortality within 30 days or during the index hospitalization, if the postoperative length of stay was longer than 30 days).

The registry was approved by the Ethics Committees of all participating centers, and informed consent was obtained from all patients.

### Data management, monitoring, and adjudication

Case report forms were sent to a central database via the Internet. Remote electronic data monitoring was performed in 100% of the cases to correct for missing and inconsistent information. On-site source document verification was randomly performed in 20% of all the included cases.

An independent committee consisting of 5 cardiologists and 1 neurologist adjudicated all adverse events.

### Statistical Analysis

The continuous variables were expressed as means ± standard deviation, and the categorical variables were expressed as percentages. The performance of the risk scores in predicting the primary outcome was analyzed through discriminative capacity (c statistic) and calibration (comparison of predicted and observed mortality rates). The capacity to discriminate between the survivors and nonsurvivors was determined using the area under the receiver-operating characteristic (ROC) curve, and the calibration was performed using the Hosmer-Lemeshow test. Plots with quartile distributions of observed and expected mortality for all scores were also presented.

The statistical software SPSS version 15.0 was used for the analyses.

## Results

In total, 418 patients were included in the registry, with a mean age of 81.5 ± 7.7 years. The median follow-up period was 343.5 days (interquartile range, 74.3-721.5). The clinical characteristics of the patients are shown in Table 1. In the population studied, 31.8% were diabetic, 78% had glomerular filtration rates (GFRs) < 60 mL/min, and 57.9% had coronary artery disease. A complete clinical follow-up was obtained from 416 (99.5%) patients.

**Table 1:** Baseline clinical characteristics

**Characteristics**	**n = 418**
Age (years)	81.5 ± 7.7
Male	200 (47.8%)
Functional Class III or IV	348 (83.2%)
Diabetes	133 (31.8%)
GFR^a^ < 60 mL/min	313 (78%)
COPD^b^	73 (17.5%)
Coronary artery disease	242 (57.9%)
Prior PCI^c^	142 (34%)
Prior CABG^d^	72 (17.2%)
Prior aortic valvuloplasty	33 (7.9%)
Prior stroke	31 (7,4%)
**Predicted 30d mortality**	
Logistic Euroscore I	20.2 ± 13.75%
Euroscore II	6.45 ± 13.75%
STS score	14.7 ± 4.38%
Ambler score	6.99 ± 3.79%
Guaragna score	17.34 ± 10.83%

a GFR: glomerular filtration rate;

b COPD: chronic obstructive pulmonary disease;

c PCI: percutaneous coronary intervention;

d CABG: coronary artery bypass graft.

TAVI was performed via transfemoral access in most patients (96.2%); CoreValve (Medtronic) was the most widely used device (86.1%). The procedure was successfully performed in 76.3% of the cases, according to the VARC definition (Table 2). The main reasons for failure were the presence of moderate to severe aortic regurgitation (9.2%), a mean residual aortic gradient ≥ 20 mmHg (4.4%), and the need for the implantation of an additional valve prosthesis (5.5%).

**Table 2:** Procedure characteristics

**Characteristics**	**n = 418**
**Access**	
Transfemoral	402 (96.2%)
Transsubclavian	09 (2.2%)
Transaortic	06 (1.4%)
Transcarotid	01 (0.2%)
**Bioprosthesis**	
CoreValve	360 (86.1%)
Sapien XT	58 (13.9%)
Successful procedure	319 (76.3%)
Failed procedure	99 (23.7%)
Moderate/severe aortic regurgitation	35/379 (9.2%)
Mean gradient ≥ 20 mmHg	16/366 (4.4%)
Additional valve prosthesis	23 (5.5%)
Surgical conversion	4 (1%)
Prosthesis malpositioning	26 (6.2%)

**Figure 1:**
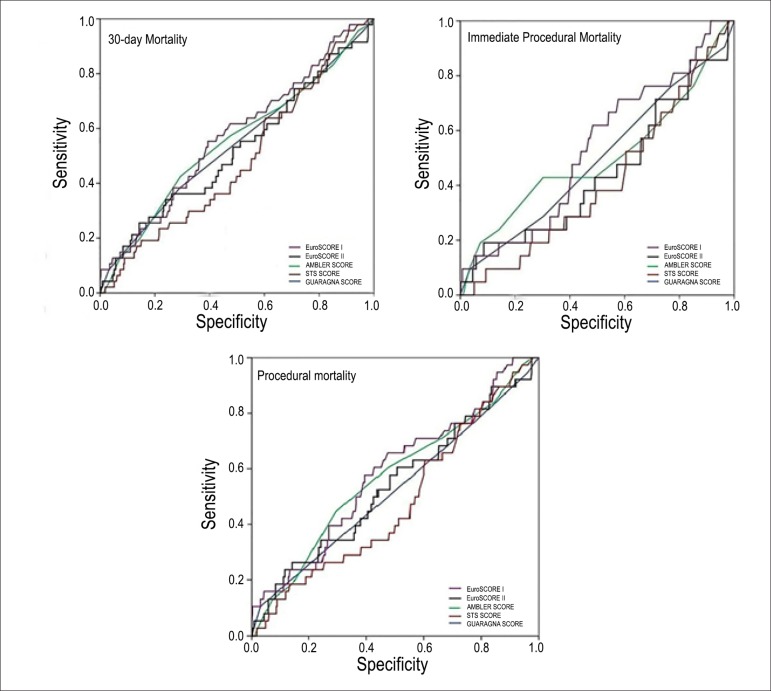
ROC curves for the outcomes assessed using the different surgical risk scores.

The overall 30-day mortality rate observed was 9.1%, the immediate procedural mortality was 5%, and the procedural mortality was 11.7%. The mortality rates predicted by the scores were as follows: logistic EuroSCORE I, 20.2 ± 13.8%; EuroSCORE II, 6.5 ± 13.8%; STS score, 14.7 ± 4.4%; Ambler score, 7.0 ± 3.8%; and Guaragna score, 17.3 ± 10.8%. The capacity to predict the 30-day mortality according to the scores is shown in Figure 1. None of the scores could accurately predict the 30-day mortality of the patients undergoing TAVI. The areas under the ROC curves were as follows: 0.58 [95% confidence interval (CI): 0.49 to 0.68, p = 0.09] for the logistic EuroSCORE I; 0.54 (95% CI: 0.44 to 0.64, p = 0.42) for the EuroSCORE II; 0.57 (95% CI: 0.47 to 0.67, p = 0.16) for the Ambler score; 0.48 (95% CI: 0.38 to 0.57, p = 0.68) for the STS score; and 0.52 (95% CI: 0.42 to 0.62, p = 0.64) for the Guaragna score (Table 3). The scores were also inadequate in discriminating between the occurrences of immediate procedural mortality and procedural mortality (Figure 1).

**Table 3:** Area under the ROC curve for 30-day mortality

**Score**	**AUC**	**95% CI**	**p-value**
Logistic EuroSCORE I	0.58	0.49-0.68	0.09
EuroSCOREII	0.54	0.44-0.64	0.42
Ambler score	0.57	0.47-0.67	0.16
STS score	0.48	0.38-0.57	0.68
Guaragna score	0.52	0.42-0.62	0.64

**Figure 2:**
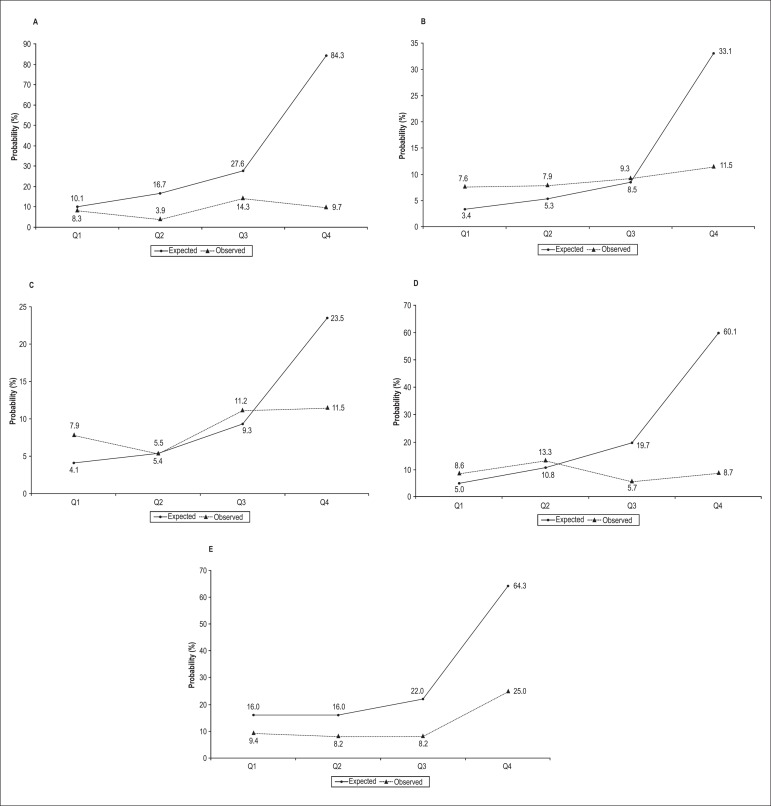
Quartile distributions of observed and predicted mortality rates according to the different surgical risk scores. A: Logistic EuroSCORE I (Q1: < 10.1%; Q2: ≥ 10.1% and < 16.7%; Q3: ≥ 16.7% and < 27.6%; Q4: ≥ 27.6%); B: EuroSCORE II (Q1: < 3.4%; Q2 ≥ 3.4% and < 5.3%; Q3: ≥ 5.3% and < 8.5%; Q4: ≥ 8.5%); C: Ambler score (Q1: < 4.1%; Q2 ≥ 4.1% and < 5.5%; Q3: ≥ 5.5% and < 9.3%; Q4: ≥ 9.3%); D: STS score (Q1: < 5.0%; Q2 ≥ 5.0% and < 10.8%; Q3: ≥ 10.8% and < 19.7%; Q4: ≥ 19.7%); and E: Guaragna score (Q1: < 16.0%; Q2 = 16.0%; Q3: > 16% and < 22.0%; Q4: > 22.0%)

All the scores exhibited good calibrations (p > 0.05) in the Hosmer-Lemeshow test. However, the logistic EuroSCORE I overestimated mortality in all quartiles when the quartile distribution of the observed and expected mortality rates was analyzed. The EuroSCORE II underestimated the mortality rates in the first and second quartiles and overestimated the mortality rates in the last quartile, although it exhibited a good calibration in the third quartile. The Ambler score underestimated the mortality rates in the first and third quartiles and overestimated the mortality rates in the last quartile, although it exhibited a good calibration in the second quartile. The STS score underestimated the mortality rates in the first and second quartiles and overestimated the mortality rates in the third and fourth quartiles. Finally, the Guaragna score overestimated the mortality rates in all of the risk quartiles (Figure 2).

## Discussion

Our study demonstrated that of the 5 different scores developed for risk assessment of patients undergoing SAVR, none could predict the 30-day mortality after TAVI.

The logistic Euroscore I overestimated the real mortality for the TAVI group in the GARY registry^14^, a German registry that enrolled 3875 patients undergoing TAVI. The low accuracy of the logistic EuroSCORE I and the STS score in predicting short-term mortality after TAVI has been demonstrated in 2 other multicenter registries: a Canadian study involving 399 patients^15^ and an Italian study that assessed 663 patients^16^. In a French study^17^, the EuroSCORE II also demonstrated low accuracy in predicting the 30-day mortality in 435 patients undergoing TAVI. In the PARTNER trial, Kodali et al. showed that the STS score was an independent predictor of mortality after SAVR but not after TAVI^18^. To our knowledge, this is the first study to evaluate the performance of the Ambler and Guaragna scores in predicting the mortality of patients undergoing TAVI.

In contrast to what has been shown in the present study, Hemmann et al^19^, through the analysis of 426 patients included in a registry involving 2 centers in Germany, showed that the STS score was a good predictor of the 30-day mortality after TAVI. The authors reported a hazard ratio of 1.06 (95% CI: 1.03 to 1.1) for each point summed in the STS score. Notably, 36% of the procedures were performed via the transapical access.

The prediction of outcomes after TAVI is a complex task. Some clinical factors, such as the presence of ventricular dysfunction, chronic obstructive pulmonary disease (COPD), cerebrovascular disease, chronic kidney failure, pulmonary hypertension, and frailty syndrome^20-22^, have been highlighted as markers of a worse prognosis. A population of patients undergoing TAVI had their frailty status evaluated through a scoring system that considered features such as weakness, malnutrition, gait speed, and degree of inactivity. Higher frailty scores were more closely associated with the 1-year mortality after prosthesis implantation^23^. Current available surgical scores do not capture some of these factors.

The surgical risk scores have limitations even in patients undergoing SAVR, probably because in a group with people of such advanced age, the features and comorbidities that may contribute to increased mortality and that are not captured by the scores are numerous. Among these features, the following stand out as independent predictors of operative mortality: frailty syndrome, hypoalbuminemia, malnutrition^24^, and previous radiotherapy to treat tumors in the chest cavity^25^.

Another explanation for the low performance of the surgical risk scores in TAVI populations is the fact that the procedures are completely different. The scores were created and validated for a major procedure that involves thoracotomy, cardioplegia, and extracorporeal circulation, with significant systemic repercussions. Therefore, the clinical characteristics that would reduce the chance of patient survival after the conventional valve replacement surgery may not have any impact on the outcomes after TAVI. Thus, the scores could overestimate the patient mortality for this procedure.

Makkar et al^26^, while analyzing patients from the PARTNER trial who were considered inoperable (cohort B), compared the influence of the technical aspects with the influence of clinical variables on the outcomes after TAVI. The authors demonstrated that patients who were deemed inoperable because of technical reasons such as a porcelain aorta, previous mediastinal radiation, chest wall deformities, and the presence of coronary grafts on sternal reentry exhibited better outcomes after undergoing TAVI than patients who were deemed inoperable because of clinical reasons.

Kotting et al^27^ developed a specific score to predict the in-hospital mortality for patients undergoing surgical or percutaneous aortic valve replacement, known as the German AV score. This score was developed on the basis of the analysis of 11,794 patients with good discriminatory performance and an area under the ROC curve of 0.8. However, as a major limitation, only 5.1% of the original population had undergone TAVI. In the GARY registry^14^, this score overestimated mortality for the TAVI group.

We estimated the calibration of observed/predicted mortality using 2 different methods. Using the Hosmer-Lemeshow test, all scores showed good calibration. However, when observing the plots of quartile distributions of predicted and observed mortality rates, we noted that their calibration was in fact poor. Recent studies have suggested that the Hosmer-Lemeshow test is imperfect and underpowered, particularly for analyzing the calibration in small samples sizes^28-30^.

The current recommendation is that the scores should be used only to identify those patients who, because of a high surgical risk, can best benefit from percutaneous therapy. Better performance for predicting the mortality after TAVI still depends on the development of specific scores for this purpose^31^. Investigators with powerful databases have already started to pursue a TAVI mortality risk. However, the accuracy obtained has been only modest, ranging from 0.59 to 0.71 (validation cohorts)^32-34^.

This study has a number of limitations. First, the data were self-reported and patient inclusion was partially retrospective. Therefore, adverse events may have been under reported. However, complete clinical follow-up was obtained from 99.5% of the patients and all adverse events were independently adjudicated. Therefore, data on survival is extremely robust. Moreover, the relatively small sample size may have precluded the detection of statistical significance.

## Conclusions

In this real-world registry, the surgical risk scores were inaccurate in predicting the mortality after TAVI. Risk models specifically developed for TAVI are required.
